# Numerical Investigation of Safety Strategy for Gas Disaster Prevention in Successive Panels Using Upper Protective Layer Mining: A Case Study

**DOI:** 10.3390/ijerph19074408

**Published:** 2022-04-06

**Authors:** Yankun Ma, Longyong Shu, Hongyan Li, Cong Cui, Yang Zhou, Yang Li

**Affiliations:** 1 China Coal Research Institute, Beijing 100013, China; yankunma@163.com; 2 Mine Safety Technology Branch, China Coal Research Institute, Beijing 100013, China; lhylhb@163.com (H.L.); cuicong521hero@163.com (C.C.); z9999pc9999@163.com (Y.Z.); 3 Xinji No. 1 Mine of China Coal Xinji Energy Co., Ltd., Huainan 236218, China; yli2022@foxmail.com

**Keywords:** coal seam group, protective layer mining, coal and gas outburst, gas control, pressure relief

## Abstract

Mine gas disasters are a major safety concern in underground coal mining. Protective layer mining is widely used in gas disaster control, but there are limited theoretical and experimental results that can provide guidance for site-specific mining circumstances. Taking the Xinji No. 1 mine as an example, gas disaster treatments were conducted in a new panel with overlying goaf located 85 m above the coal mine and adjacent goaf located at 30 m intervals. This study involved a comprehensive investigation, which included four steps: the selection of the first mining face, gas control and prevention, tracking and investigation, and effect analysis and assessment. The safety strategy focused on gas control planning in new mining areas or panels. The distribution and evolution characteristics of the stress, the gas permeability coefficient and the deformation volume within the protected layer were determined by numerical simulation. The coal deformation, gas emission and extraction effect were analyzed by field observation. The deformation and gas permeability of the coal seam were consistent with the stress evolution, for which the maximum compressional and expansional deformation of 6-1 coal were 18‰ and 28‰, respectively. Gas disaster control and prevention treatment of the mining face produced a significant protective effect on the underlying No. 6-1 coal seam. This work is beneficial for the planning of gas control in successive panels.

## 1. Introduction

China is the world’s largest energy consumer. China’s primary energy consumption, of which coal accounted for 56.8% in 2020, will reach its peak in 2030 [1]. Coal resources play a long-term role in China’s energy security. Overall, 70% of coal-production districts in China are characterized by the occurrence of gassy coal seam groups [2]. The mode of coal seam group mining for gas disaster prevention has been well applied in China’s mining fields, including the Beipiao, Nantong, Songzao, Tiefa, Huainan, Xishan and Shuangliu mining areas. The long-wall mining method is widely used in China, and huge goaf areas are formed during the mining process, which produces great disturbance effects. Mining effects, such as rock strata movement, stress transfer, pressure relief and stress concentration, lead to the release and migration of gas from adjacent coal seams [3,4]. Utilizing the mining effects produced by the priority coal seam, gas extraction technology is applied to prevent the occurrence of gas accidents [5,6].

With the advancement of coalfaces, coal seams are expected to undergo a cycle of stress evolution that involves in situ stress (stress abutment), failure and stress reduction (stress recovery). Similarly, the evolution in the permeability of coal during protective layer mining was described experimentally by Yin et al. in four stages [7]. The decrease in coal permeability can increase the risk of gas-dynamic phenomena [8]. The permeability of intact coal and post-failure coal were formulated by Durucan et al. and it was shown that the latter one is less sensitive to stress [9,10]. Connell [11] developed a coupled model by assuming uniaxial triaxial strain and constant vertical stress. Liu and Rutqvist [12] developed a permeability model for the condition of uniaxial strain and constant confining stress. Exponential relationships between the coal permeability and axial strain during the loading and unloading stages were proposed by Chen et al. [13]. However, mechanical conditions at the local scale are much more complex in coal seams. In China, the permeability coefficient of the coal seam is measured in situ and used in most collieries. This parameter, which reflects the resistance of the gas flow in the coal seam, was defined by Zhou based on the uncoupled gas flow law in the coal mass [14]. On the basis of the measured borehole gas flow at underground atmospheric pressure, the permeability coefficient of coal seam is calculated using a series of formulas [15].

The effect of the protective layer on mining is related to parameters such as layer spacing, dip angle and lithology of adjacent coal seams, and its protective effect needs to be systematically investigated [16]. Guo et al. [17] investigated rock strata movement, fracture development and goaf permeability in relation to coal mining, which help the practice of the concept of coal and gas co-mining. Xu et al. [18] investigated gas disaster control in conditions in which there was a thick-hard roof and an igneous intrusion area. Wang et al. [19] proposed a safety strategy using a soft rock protective layer for coal mining and gas outburst prevention. Cheng et al. [20] developed protective layer mining technology and formed a benign green coal mining mode; this was achieved by integrating gas prevention for the workface, gas extraction on the surface, and methane utilization technology. With the development of the coal industry, shallow coal resources have been depleted and the mining depth has gradually extended [21]. Stress, gas content and pressure tend to increase with the mining depth, and low gas permeability results in poor gas extraction.

Gas content is used worldwide by coal mines to assess outburst risk [22,23]. Conventional pre-drainage cannot effectively reduce the gas content of the coal seam below the threshold levels. Control methods for the pre-drainage of the seam, including surface boreholes, water infusion, hydraulic fracturing, directional drilling and pre-working a protective seam, have been suggested by scholars [23,24,25]. Compared with gas prevention treatments such as hydraulic punching and advanced drilling extraction, protective coal seam exploitation is the most effective method to treat deep gas disasters [26,27,28].

Protective layer mining is widely used in gas disaster control, but limited theoretical and experimental results can provide guidance for site-specific mining circumstances. A new panel in a deep mining area entails the facing of more severe challenges in gas disaster prevention. Thus, the evaluation of gas occurrences and the investigation of mining disturbances in protected coal seams, including evolutions of stress, coal body deformation, and permeability, are the basis for safe mining activities, such as mining engineering design in new areas or levels, and reasonable short-term gas control planning. Taking the Xinji No. 1 mine as an example, a set of systematic investigation plans has been put forward to eliminate the outburst danger and ensure the safe mining of the workface. In addition, the spatial variability of gas permeability in coal seams with protective layer mining is presented through simulation analysis in this paper.

## 2. Investigation Scheme and Details

**Figure 1:**
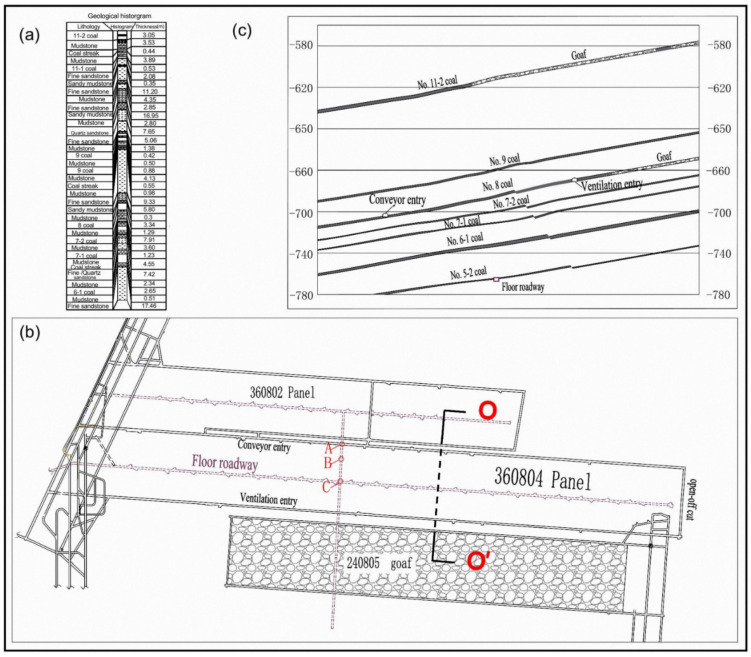
Geological condition of workface: (**a**) geological histogram; (**b**) layout of workface; (**c**) longitudinal section of line O-O’. (‘A’ and ‘C’ represent the site of gas content and pressure measuring. ‘B’ represents the site of coal deformation observing).

Xinji No. 1 mine is located in Huainan coalfield, Anhui Province, China, with an annual output of 1.8 million tons. With the geological characteristic of the group occurrence of high-gas coal seams, No. 13-1 and 6-1 coal is in danger of outburst disasters due to the high gas content, and other No. 9, 11-2, 8 coal seams, etc., all have gas occurrences. No. 8 coal seam is extracted in a new mining area (3608), where the coal seam dip angle is 3~15° and its thickness ranges from 2.0 to 4.6 m. The depth of the No. 8 coal seam in the mining area is from 695 m to 810 m, and the No. 6-1 coal seam is from 735 m to 850 m. The layout and histogram of the workface in the mining area are shown in Figure 1. The average oblique length of the workface is 180 m, and the length in the strike direction is 1339 m. The spacing between the No. 8 coal and the overlying No. 9 and 11-2 coal seams is 20 m and 85 m, respectively. The spacing between the No. 8 coal and the underlying No. 7 and 6-1 coal seams is 10 m and 28 m, respectively. 

**Figure 2:**
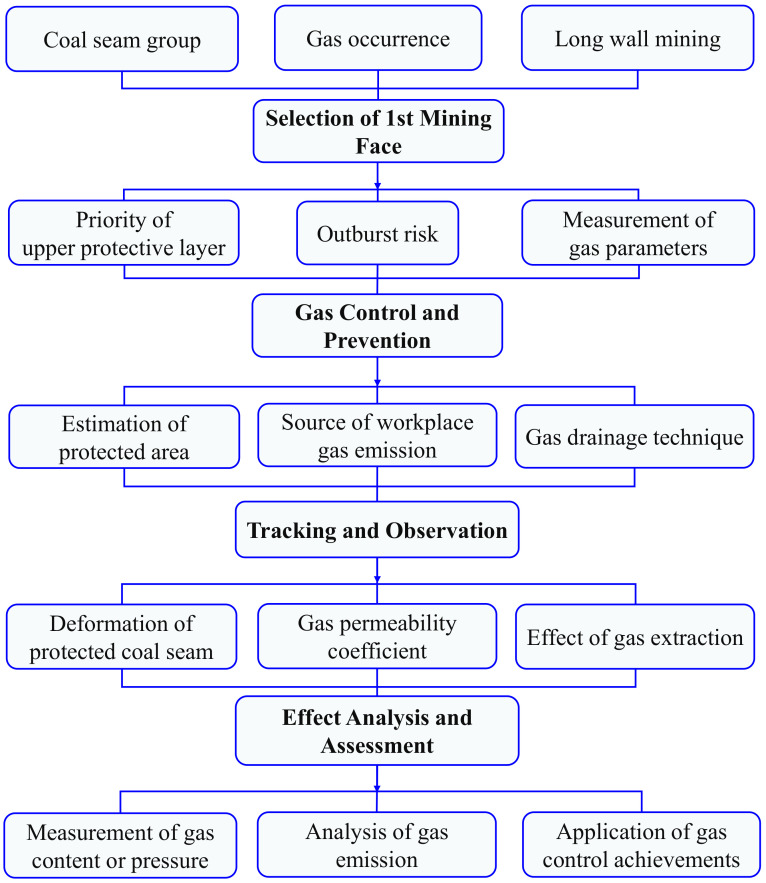
Investigation scheme of mining face in protective coal seam.

A flow chart for the investigation of the protective layer is shown in Figure 2. It goes through four stages as follows: Selection of First Mining Face, Gas Control and Prevention, Tracking and Observation, and Effect Analysis and Assessment.

### 2.1. Selection of First Mining Face

The gas occurrence conditions of the coal seam group were evaluated initially, and priority coal seam was selected as the protective layer. The gas parameters for each coal seam are shown in Table 1. No. 8 coal seam was identified as the coal seam without outburst danger by authorized research institutions. No. 6-1 coal seam, which once had dynamic incidents in the coal field, was considered to be associated with outburst danger.

No. 8 coal, with a small gas hazard level, was selected as the upper protective layer. The main reasons were as follows: firstly, the upper protective layer was preferred for mining, as it produces a pressure-relief effect and does not damage the coal seam as a result of rock strata movement; secondly, compared with No. 6-1 coal, No. 8 coal has less potential risk of gas disaster, and has better economic benefits from the perspective of resource reserves. The location of the first mining face is usually selected where geological conditions are representative or gas disaster is easy to be controlled.

The 360804 workface, with the overlying goaf located above and the adjacent goaf located nearby the coalmining area, was selected as the first mining face. The overlying goaf formed as a result of the No. 11-2 coal mine in 2011, and is located above the No. 8 coal mining area. The adjacent goaf was formed as a result of the No. 8 coal mine to the south of the mining area in June 2007, and is blocked by a coal pillar of 30 m width. The adjacent and overlying goaf produce a pressure-relief effect that can be conducive for gas control in the 360804 workface.

### 2.2. Gas Disaster Control and Prevention

A predictive method based on different gas sources was used to calculate the gas emissions in the process of coal mining, including the gas emissions from the mining seam and the adjacent seam (refer to the Safety standard of China [29]). The calculation formulae are as follows:

(1) $$q=q_{1}+q_{2},$$

(2) $$q_{1}=K_{1}\cdot K_{2}\cdot K_{3}\cdot \left(w_{0}+w_{c}\right)\cdot m/M,$$

(3) $$q_{2}=\sum_{i=1}^{n}\left(w_{0i}+w_{ci}\right)\cdot \eta_{i}\cdot m_{i}/M,$$

where *q* is the relative gas emission rate in the coalface, m^3^/t; *q*_1_ and *q*_2_ are the gas emission rates in the mining seam and the adjacent seam. *K*_1_, *K*_2_ and *K*_3_ are the rock emission coefficient, coal drop coefficient and preparatory workings discharge coefficient, respectively. *m* and *M* are the seam thickness and mining height. *w*_0_, *w*_0*i*_, *w_c_* and *w_ci_* are the original gas content of the mining coal seam, the original gas content of the *i*th adjacent seam, the residual gas content of the mining coal seam, and the residual gas content of the *i*th adjacent seam, respectively, m^3^/t. *η_i_* is the emission ratio of the *i*th adjacent seam, %. All parameters were selected as shown in Table 2. It was calculated that *q*_1_ is 1.8 m^3^/t, *q*_2_ is 2.6 m^3^/t and *q* is 4.4 m^3^/t.

**Figure 3:**
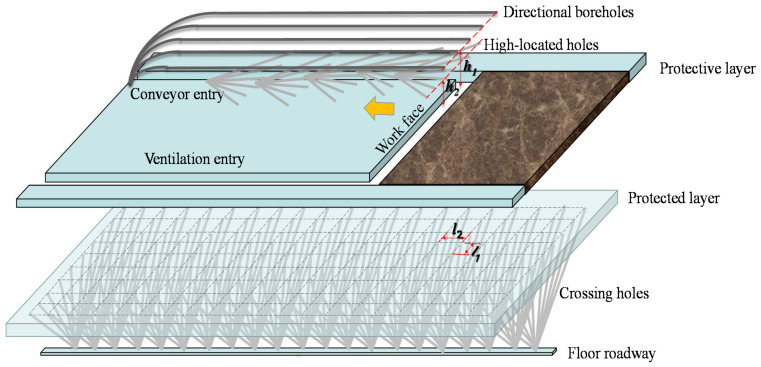
Gas extraction pattern in the mining face (*h*_1_ = 40 m, *h*_2_ = 10–23 m, *l*_1_ = *l*_2_ = 20 m).

Gas control in the mining face mainly included reducing the gas flooding into the workface and eliminating outburst disasters in the protected layer. For the first mining face, a conservative method was adopted for gas disaster control. The post-drainage technology [30,31] was adopted to prevent the gas explosion area from moving to the workface. Two kinds of borehole arrangements, high-level boreholes and directional boreholes, were adopted as gas control methods, as shown in Figure 3. A drilling field of 5 directional boreholes was arranged every 500 m in the conveyor roadway, and the bottom of the drilling area was located 40 m above the roof. A drilling field of 6 high-level boreholes was arranged every 40 m in the conveyor roadway, and the bottom of the drilling area was located 10~23 m above the roof.

**Figure 4:**
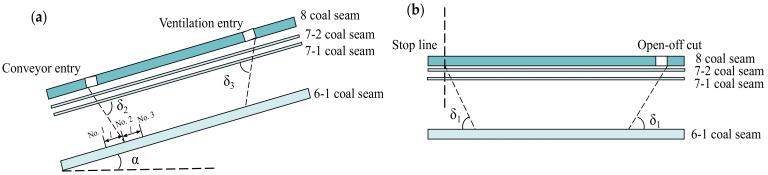
Pressure-relief angle for 6-1 coal seam: (**a**) dip-pressure-relief angle; (**b**) strike-pressure-relief angle. (No. 1, No. 2 and No. 3 are the measurement points of residual gas pressure and content. *l* = 15 m).

As calculated above, the gas emission from adjacent coal seams accounted for a large proportion of the overall emissions. Thus, the outburst disasters of the 6-1 coal seam needed to be considered and controlled. Determination of the pressure-relief range for 6-1 coal, described by the pressure-relief angle, was an important link, as shown in Figure 4. In the pressure-relief region, the gas content could be effectively reduced to less than the threshold of 8 m^3^/t by coal bed degassing. It could preliminarily be obtained by empirical method and numerical simulation method, and then determined by field verification method. The empirical method mainly refers to Appendix [16] of the Rules for Prevention and Control of Coal and Gas Outburst. The pressure-relief angle in the dip direction was selected as 75°. The strike-pressure-relief angle, located at the starting line or the stopping line, was selected as 56°, while the stoppage time of the protective layer coalface was more than 3 months and the pressure relief was sufficient. Numerical simulation methods and field validation are presented in Section 3 and Section 4.

The most fundamental method of gas disaster control is gas extraction. Utilizing the mining effect of the No. 8 coal seam, the pre-drainage technique assisted in the coal mining process by reducing the gas escaping to the workface above and eliminating the outburst risk for the No. 6-1 coal seam. The crossing holes were prepared for drainage of methane and fully covered the vertical projection range of the 360804 workface. The interval between the hole bottoms was 20 m in the transverse and longitudinal directions, as shown in Figure 3.

### 2.3. Tracking and Observations

The research on the mining effect mainly includes the mine’s ground pressure, the gas emission of the coalface, the deformation of the protected coal body, the permeability coefficient of the protected layer, the extraction effect, etc.

#### 2.3.1. Observation of Coal Deformation

**Figure 5:**
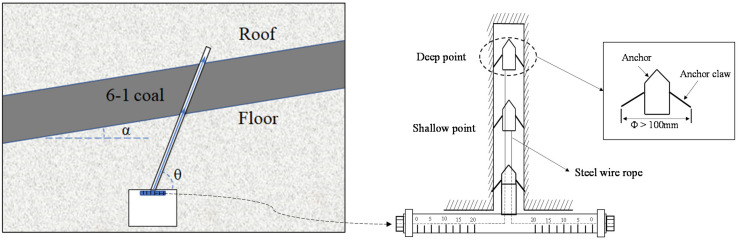
Installation diagram of multi-point displacement meter.

Observation of the deformation of the coal body was carried out in the floor roadway. The crossing hole of No. 6-1 coal was constructed to install the multi-point displacement meter, as shown in Figure 5. The deformation of volumetric strain increase was calculated using Equation (4). The test location was at the pressure-relief boundary of the investigated area in the dip direction, as shown in Figure 1 (site B). The azimuth of the hole was 276°, the dip angle was 63° and the hole length was 33.5 m.

(4) $$\eta =sin\left(\theta +\alpha \right)\cdot \left(l_{1}-l_{2}\right)/H\times 1000,$$

where η is the volumetric strain increase in the coal body, ‰; *θ* is the installation drilling angle, °; *α* is the dip angle of the coal seam, °; *l*_1_ and *l*_2_ are the displacements of the deep and shallow base points, m; *H* is the thickness of the coal seam, m.

#### 2.3.2. Permeability Coefficient Test

The gas permeability coefficient was used to characterize the resistance of the gas flow in the coal seam and the coefficient reflected the difficulty of gas drainage in the coal seam, in units of m^2^/(MPa^2^·d). A crossing hole was drilled into the coal seam from the roadway. The borehole was sealed and equipped with a pressure gauge until the values of the gas pressure were stable. After the pressure gauge was removed, the gas flow was observed with a flowmeter within a few days. The stable rate of flow was used to calculate the permeability coefficient [15]. The test result for original permeability coefficient $\lambda_{0}$ was 0.093 m^2^/MPa^2^·d. The relationship between the permeability coefficient and coal permeability is expressed as follows:

(5) $$\lambda =k/\left(2\mu p_{n}\right),$$

where λ is the permeability coefficient of the coal seam, m^2^/(MPa^2^·d); *k* is the permeability of the coal seam, m^2^; *μ* is the dynamic viscosity, Pa·s; *p_n_* is the atmospheric pressure, Pa. Due to pressure relief and degassing, the *λ* of No. 6-1 coal in the condition of coal mining was difficult to measure but it could be investigated by numerical simulation (Section 4).

#### 2.3.3. Observation of Gas Emission and Extraction

Gas emission and extraction in the mining face can be observed using a gas monitoring system. The flow rate of the gas drainage and the gas concentration of the return air can indicate whether the disaster treatments are effective. There is a significant relationship between the mining pressure behavior and the gas emission in the stope, and one of the important indexes is the support resistance, which represents the strata movement.

### 2.4. Effect Analysis and Assessment

The mining effect was observed and investigated until the end of the mining activities in the workface. Thus, the residual gas content and pressure were tested for the protected layer. The gas content and gas pressure were used to evaluate the outburst risk in the coal seam. A gas content of 9 m^3^/t is used as the threshold value in Australia, while 8 m^3^/t or 0.74 MPa of gas pressure is used in China. Test results, in combination with observation results, were used for analyzing whether the treatments for gas control reached the expected effects. Based on the whole-process investigation, a reasonable and grounded scheme for gas disaster control can be applied to adjacent workfaces or others with similar conditions.

## 3. Numerical Modeling

### 3.1. A Stability Assessment Model for Coal and Rock

#### 3.1.1. Strain-Softening Mohr–Coulomb Model

A strain-softening model was employed by Wang et al., Esterhuizen et al. and Sherizadeh and Kulatilake for coal and rock masses [32,33,34]. The combination of elasto-plastic behavior and the post-failure response of the material can present the whole stress–strain process. The strain-softening model available in FLAC3D (modelling software) is a composite Mohr–Coulomb criterion with tension cutoff. The Mohr–Coulomb non-associated elasto-plastic model describes the conditions of shear failure of rock and coal:

(6) $$f=\sigma_{1}-\sigma_{3}N_{\varphi }+2C\sqrt{N_{\varphi }}\geq 0,$$

where $\sigma_{1}$, $\sigma_{2}$ and $\sigma_{3}$ are the maximum, intermediate and minimum principal stress, respectively, MPa; *φ* is the friction angle, °; *C* is the cohesion, MPa; $N_{\varphi }=\left(1+sin\varphi \right)/\left(1-sin\varphi \right)$.

The tension failure of coal and rock is described by the following criterion:

(7) $$f_{t}=\sigma_{3}-\sigma_{t}=0,$$

where $\sigma_{t}$ is the tension strength, MPa.

The flow rule for plastic yielding has the following form:

(8) $$\varepsilon_{ij}^{p}=\lambda \;\partial g/\partial \sigma_{ij},$$

The potential function for shear yielding is $g^{s}$, which corresponds to the non-associated law:

(9) $$g^{s}=\sigma_{1}-\sigma_{3}N_{\psi }\;,$$

The potential function for tensile yielding is $g^{t}$, which corresponds to the associated law:

(10) $$g^{t}=-\sigma_{3}\;,$$

where $\varepsilon_{ij}^{p}$ is the plastic strain tensor; λ is a constant; ψ is the dilation angle, °; $N_{\psi }=\left(1+sin\psi \right)/\left(1-sin\psi \right)$.

The friction and cohesion may harden or soften after the onset of plastic yield:

(11) $$\varphi =\varphi_{e0}+\left(\left(\varphi_{ef}-\varphi_{e0}\right)E_{eq}^{p}\right)/\left(B_{p}+E_{eq}^{p}\right),\;C=C_{0}+\left(\left(C_{f}-C_{0}\right)E_{eq}^{p}\right)/\left(B_{c}+E_{eq}^{p}\right),$$

where $\varphi_{e0}$ and $C_{0}$ are elastic initial values, and $\varphi_{ef}$ and $C_{f}$ are plastic limit values in the plastic regime. $B_{p}$ and $B_{C}$ are hardening/softening parameters. $E_{eq}^{p}$ is the equivalent plastic strain defined by a measure of the second invariant of the plastic shear-strain increment tensor:

(12) $$E_{eq}^{p}=1/\surd 2\;\surd ({\left(\Delta \varepsilon_{1}^{p}-\Delta \varepsilon_{m}^{p}\right)}^{2}+{\left(\Delta \varepsilon_{m}^{p}\right)}^{2}+{\left(\Delta \varepsilon_{3}^{p}-\Delta \varepsilon_{m}^{p}\right)}^{2}\;),\;\Delta \varepsilon_{m}^{p}=\frac{1}{3}\;\Delta \varepsilon_{1}^{p}+\Delta \varepsilon_{3}^{p})\;,$$

where $\Delta \varepsilon_{1}^{p}$ and $\Delta \varepsilon_{3}^{p}$ are incremental plastic strains that correspond to the maximum and minimum principal stress.

The plastic tensile-strain increment $E_{eq}^{pt}$ is expressed as follows:

(13) $$E_{eq}^{pt}=\left|\Delta \varepsilon_{3}^{p}\right|,$$

#### 3.1.2. Double-Yield Model

The fallen rock in goaf presents irreversible compaction and hardening behavior with the application of isotropic pressure. The double-yield model embedded in FLAC3D is a well-accepted criterion that was used to describe the volumetric yield and strength failure by Wang et al., Yavuz and Jiang et al. [32,35,36]. A volumetric yield surface is defined by the cap pressure, activated by volumetric plastic strain. As goaf materials become more compact, their elastic and plastic stiffness increases. The relation between elastic stiffness and strain is expressed by Hooke’s law. The plastic stiffness is considered according to the cap pressure. Using incremental notation, the law is defined by relation as follows:

(14) $$K_{c}=min\left(R\Delta p_{c}/\Delta e^{pv},\;K\right),$$

(15) $$G_{c}=G\;K_{c}/K,$$

where $K_{c}$ and $G_{c}$ are the current tangential bulk and shear moduli, GPa. *R* is a constant factor. $p_{c}$ is the cap pressure and $\Delta e^{pv}$ is the plastic volumetric strain expressed with the following equation:

(16) $$\Delta e^{pv}=\Delta e_{1}^{pv}+\Delta e_{2}^{pv}+\Delta e_{3}^{pv},$$

The volumetric yield function $f^{v}$ is defined by the following equation:

(17) $$f^{v}=1/3\;\left(\sigma_{1}+\sigma_{2}+\sigma_{3}\;\right)+p_{c}\;$$

#### 3.1.3. Permeability Evolution Equation

As mining stress is coupled with the gas transport in the coal seam, the evolution of the coal permeability during coal mining is described by permeability models under conditions of variable stress [37]. Protected coal seams experience complex stress variation during protective seam mining. The sensitive relationship between the permeability of the coal seam and the stress variation is expressed empirically by Equation (18) [38,39]. The change in the mining-induced stress is described by the difference of the first stress invariant:

(18) $$\lambda =\lambda_{0}\;exp\left[-\gamma \left(J_{\sigma }-J_{\sigma 0}\;\right)\right],\;J_{\sigma }=\sigma_{1}+\sigma_{2}+\sigma_{3}\;,$$

where $J_{\sigma }$ and $J_{\sigma 0}$ are the first effective stress invariants and the initial first effective stress invariants, respectively, in MPa; *γ* is the coefficient related to stress.

### 3.2. Global Model and Simulation Plans

**Figure 6:**
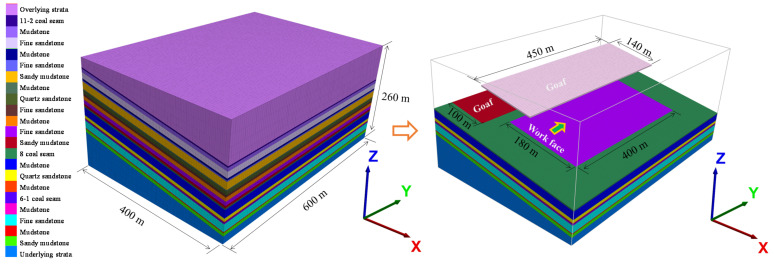
Schematic diagram of numerical geometric model.

A global model with a three-dimensional size of 400 × 600 × 260 m in the x, y and z directions, respectively, was constructed by FLAC3D, as shown in Figure 6. The horizontal displacements of the model, four vertical side faces, were restricted in the normal direction, and the vertical displacement at the base of the model was set at zero. The vertical and horizontal stresses applied in the model were calculated by Equation (19), derived from in situ hydraulic fracturing stress test results [40]:

(19) $$\left\{\begin{array}{c} S_{H}=0.062H-15.91\\ S_{h}=0.038H-4.600\\ S_{V}=0.033H+0.308 \end{array}\right.\;$$

where *H* is the buried depth, m; $S_{H}$, $S_{h}$ and $S_{V}$ are the maximum horizontal earth stress, minimum horizontal earth stress and vertical earth stress, respectively. A stress of 16.8 MPa was applied on the top of the model to simulate the overburden weight. Stresses of 22.4 MPa in the horizontal X direction and 28.4 MPa in the Y direction were applied on the vertical faces of the model. The range of the overlying goaf was 140 × 450 m, that of the adjacent goaf was 100 × 450 m, and that of the 360804 workface was 180 × 360 m with a tilt angle of 9° (Figure 6).

The physical and mechanical rock mass parameters were obtained via laboratory tests of specimens together with an empirical conversion [41], as shown in Table 3. The rock strata were described using the Mohr–Coulomb model, the coal seams via the strain-softening model, and the goaf materials by means of the double-yield model. The strain-softening model allowed the representation of the softening behavior of nonlinear material based on the Mohr–Coulomb model’s properties. The softening behaviors are specified in Table 4. In the double-yield model, the relation between pressure and volumetric change, as expressed in Table 5, involved relating the cap pressure to the plastic volume strain. Volumetric yielding occurred when there was isotropic stress at the cap pressure. Parameter values in Table 4 and Table 5 were estimated from a review of the parameter values used for the modeling of similar problems [32,33,34,35,36,37].

**Figure 7:**
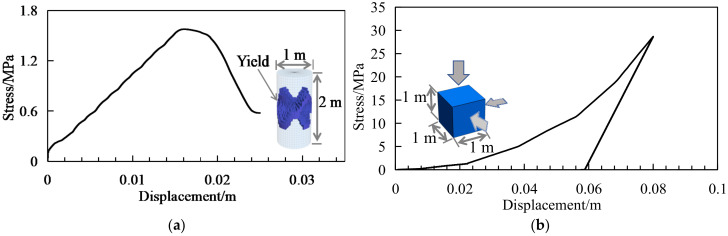
Simulated curves for specimens: (**a**) uniaxial compression results of coal sample; (**b**) triaxial test of goaf material sample.

A uniaxial compression test of a coal sample, with a height-to-diameter ratio of 2, was simulated as shown in Figure 7a. The axial loading on the sample was at a constant velocity of 10^−6^ m/s. The stress–strain curve showed obvious pre-peak hardening and post-peak softening behaviors. An “X-shape” yield surface was produced in the sample during loading. A cube element of goaf material with triaxial loading and unloading was simulated as shown in Figure 7b. Triaxial loading on the sample was at a constant velocity of 10^−6^ m/s and the triaxial unloading was 0.1 m/s. With the increasing of the stress, the sample volume was compressed, and the volume could not be completely recovered during unloading. The loading and unloading curve present the volumetric yielding behavior.

Numerical simulation was carried out in the following steps: firstly, No. 11-2 coal above the 360804 workface was excavated to simulate the formation of the overlying goaf; secondly, No. 8 coal next to the 360804 workface was excavated to simulate the formation of the adjacent goaf; thirdly, the 360804 workface was mined out gradually. The mining effect was analyzed during excavation, including the stress evolution, gas permeability distribution and coal deformation.

### 3.3. Distribution of Stress and Gas Permeability Coefficient in No. 6-1 Coal

**Figure 8:**
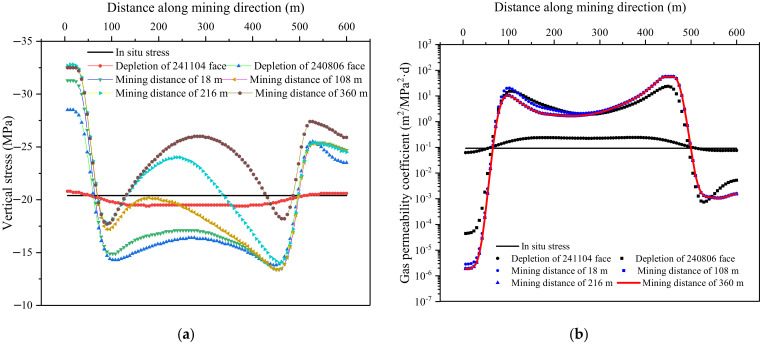
Stress and gas permeability coefficient curves within 6-1 coal seam: (**a**) vertical stress; (**b**) gas permeability coefficient.

**Figure 9:**
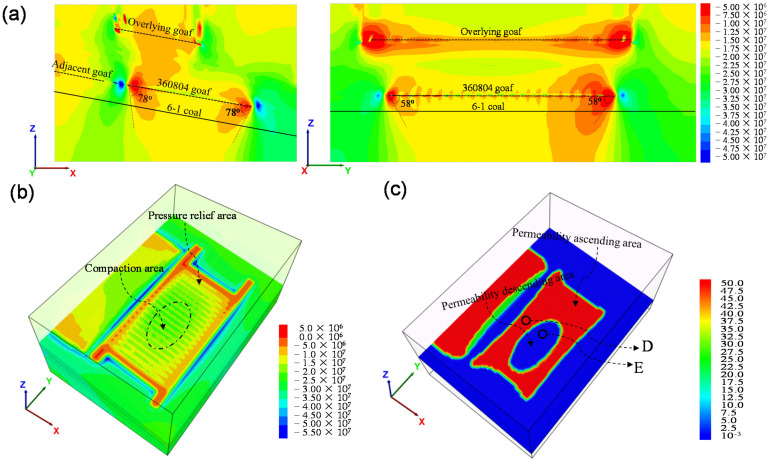
Distribution of stress and gas permeability coefficient: (**a**) slices of maximum principal stress in strike and dip directions (unit: Pa); (**b**) vertical stress distribution in stope (unit: Pa); (**c**) distribution of gas permeability coefficient of 6-1 coal (unit: m^2^/MPa^2^·d). (D and E are observing points).

Curves representing the stress and gas permeability coefficient within the No. 6-1 coal seam were used to describe mechanical response during mining (Figure 8). The overlying goaf led to a decreasing of the vertical stress by about 2 MPa within the underlying 6-1 coal. The adjacent goaf had a significant influence on the stress distribution of workface 360804 and the maximum gas permeability coefficient was 25.3 m^2^/(MPa^2^·d). For the mining of the 360804 workface, a compaction area was formed in the middle of the goaf, where the stress was restored and the gas permeability was gradually reduced. Figure 9a shows the distribution of the maximum principal stress along the strike and dip when the workface was stopped for 360 m. The pressure-relief range of the No. 6-1 coal under the workface was greater than the region defined using the empirical method, where the pressure-relief angles in the dip and strike direction were redefined as 78° and 58°, respectively. The pressure-relief range in the goaf formed an “O-shape” distribution and the high permeability coefficient within the 6-1 coal also presented an “O-shape” distribution, as shown in Figure 9b,c. The maximum permeability coefficient was 55.5 m^2^/(MPa^2^·d), which was 600 times that of the original state.

### 3.4. Evolution of Mining Disturbance for No. 6-1 Coal

**Figure 10:**
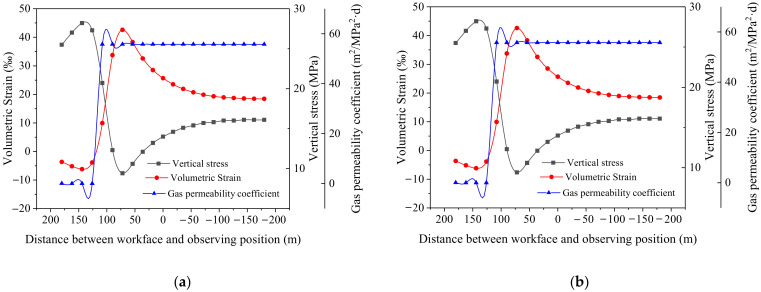
Evolution of stress, volume strain and gas permeability coefficient: (**a**) point D; (**b**) point E.

Point D and E were chosen to present the evolution of mining disturbances for 6-1 coal (Figure 9c). With the variation of the abutment stress in the workface, the stresses within the No. 6-1 coal changed. When the distance between the coalface and the observation point was 140 m, the coal stress reached its maximum value, the deformation reached its minimum value, and the permeability coefficient reached its minimum value, as shown Figure 10. As the stress decreased, the deformation volume and permeability coefficient within the coal increased. The volume deformation and permeability coefficient tended to be stable when the compacted area was formed in the goaf above. When the abutment stresses in the compaction area recovered, the deformation volume and gas permeability coefficient decreased. The No. 6-1 coal in the “O-shape” area maintained a higher gas permeability coefficient, while in the compaction area, there was a reduced gas permeability coefficient, but it was higher than that of the initial state.

## 4. Field Application and Analysis

**Figure 11:**
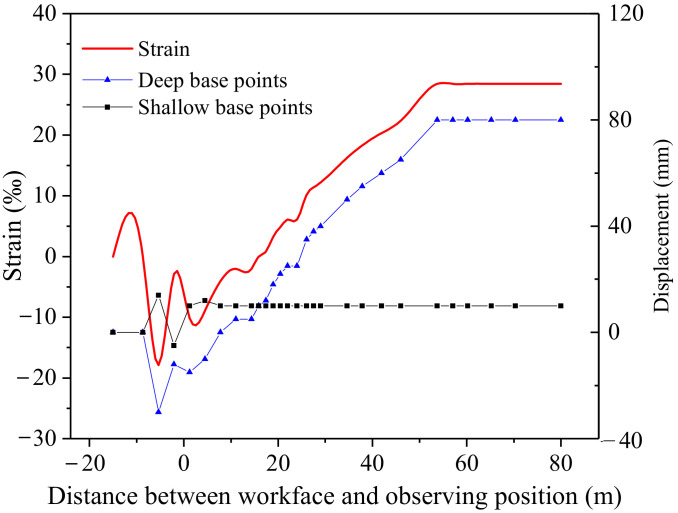
Deformation curves of 6-1 coal.

The results of the field observation of the No. 6-1 coal deformation are shown in Figure 11. As the mining disturbance effect increased, the coal body was compressed and then the pressure was released rapidly. The displacements of the deep point and the shallow point decreased while the mining face was approaching the observation site. The displacement of the deep point went up and finally stabilized at 80 mm with the mining face away from the observation site. The shallow point was inactive and the final displacement was 10 mm. Based on Equation (4), the maximum compressional and expansional deformation were 18‰ and 28‰, respectively. They were similar to the numerical modeling results in that the maximum compressional and expansional deformation were 6.2‰ and 18‰, respectively.

**Figure 12:**
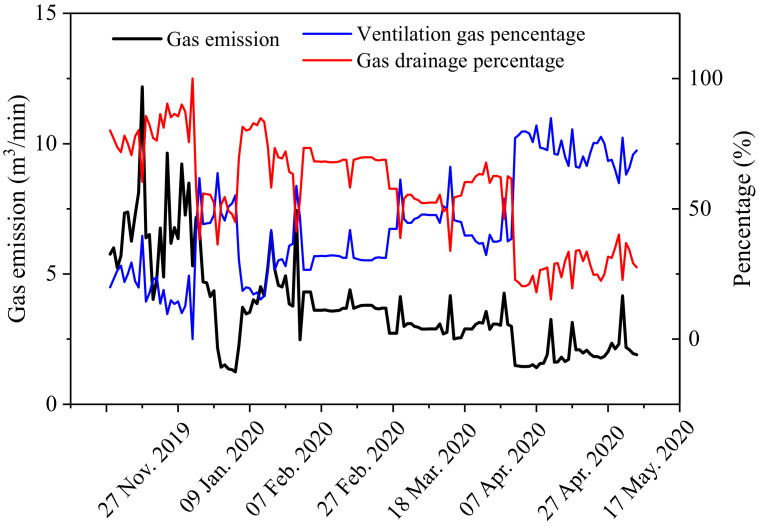
Variation of gas emission during coal mining.

The variation of the gas emission in the workface is shown in Figure 12. The gas emission volume was large in the first two months and about 80% of the released gas entered the gas-drainage system. Then, the gas emission gradually decreased with the coal mining. This was mainly due to the large amount of gas released by No. 6-1 and No. 7 coal during the initial pressure-relief period. As the gas content of the No. 6-1 coal seam was reduced with the gas extraction, the volume of gas emitted to the workface decreased correspondingly.

**Figure 13:**
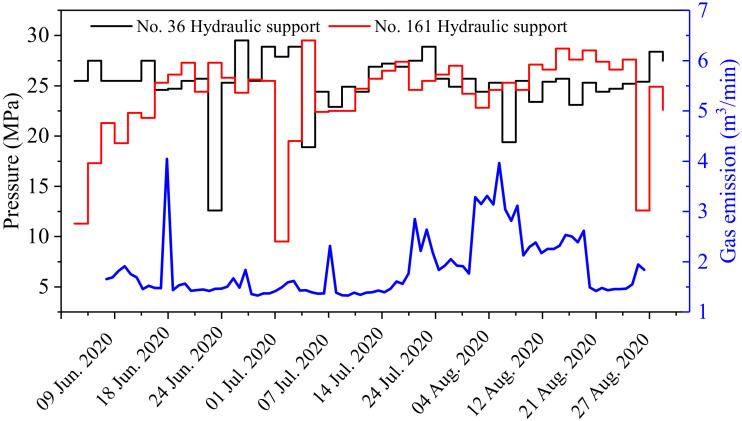
Gas emission and hydraulic support pressure curves.

In the process of mining, there was a significant relationship between the strata behavior and the gas emission. Figure 13 shows the variation of hydraulic support pressure in the workface with the gas emission. With the increase in the pressure of the hydraulic support, the amount of gas emission increased significantly. This was because the obvious movement of the rock strata led to a change in the gas flow parameters in the goaf, resulting in the movement of the explosive gas zone towards the workface. These results are similar to the research results in the literature [42,43].

**Figure 14:**
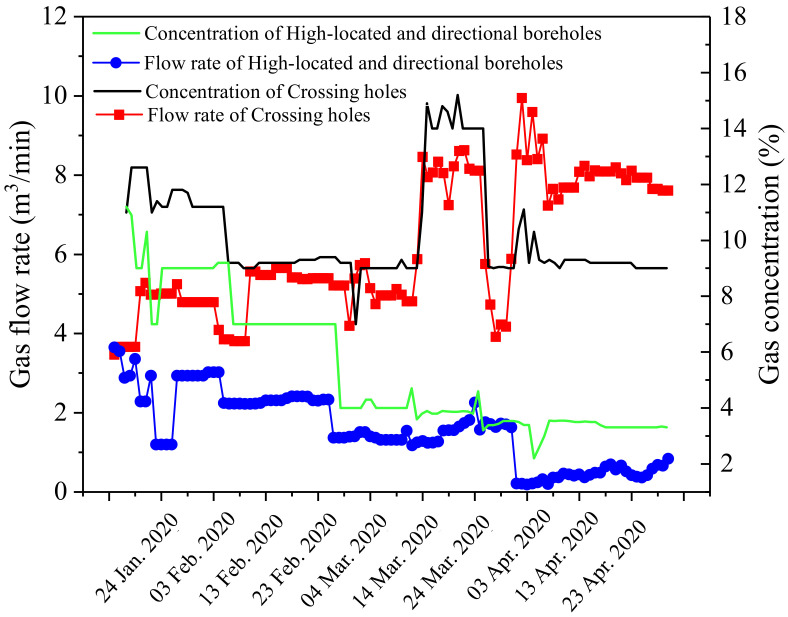
Gas flow rate and concentration.

Variation curves for the gas extraction quantity and concentration are shown in Figure 14. With the increase in the mining disturbance, the gas flow rate and gas concentration caused by the crossing holes increased, and these were stable at 8 m^3^/min and 9%, respectively. With the post-drainage technology adopted on the workface, the gas flow rate and gas concentration caused by high-level drilling and directional drilling gradually decreased from 3.8 to 2 m^3^/min and from 11% to 3%, respectively. It can be concluded that pre-drainage of No. 6-1 coal is the key to preventing gas emission in the workface and controlling outburst disasters.

After the stopping of the 360804 coalface (about one year), the gas content and pressure within the No. 6-1 coal were tested and the measurement points were located at A and C in Figure 1. Test results are shown in Table 6 and the locations of the No. 1 to No. 3 boreholes at the boundary are shown in Figure 4a. Within the range prescribed by the dip-pressure-relief angle of 78° and the strike-pressure-relief angle of 58°, the gas content of the No. 6-1 coal was reduced to 2 m^3^/t, and the gas pressure was 0.1 MPa. The treatments for the outburst disaster of the No. 6-1 coal seam were very effective.

## 5. Discussion

By conducting a comprehensive investigation process for the 360804 workface, the regularity of mining disturbances was well described, especially the variation of the gas emission and the evolution of the permeability coefficient. With the overlying goaf and the adjacent goaf, the coal mining had a remarkable pressure-relief effect on the underlying No. 6-1 coal. The important parameter values, namely the pressure-relief angles in dip and strike direction, were obtained to describe the pressure-relief range. The risk of outburst disaster in the No. 6-1 coal seam and gas emission in the mining face were controlled effectively. According to the evolution of the 6-1 coal deformation and the permeability coefficient, the control efficiency of gas disasters was improved with the increasing of the scope of mining disturbance. The occurrence of gas within the scope of the mining disturbance was conducive to outgassing.

**Figure 15:**
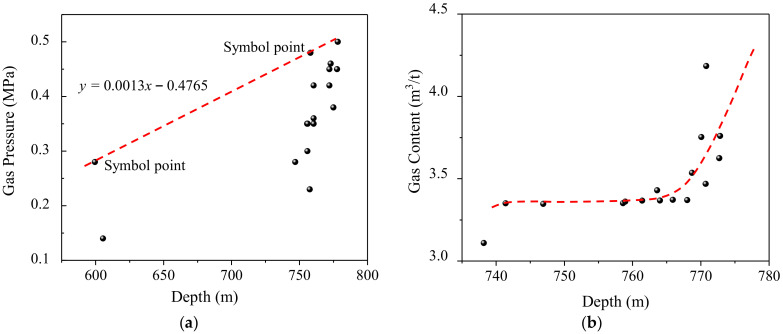
Prediction of gas occurrence within 6-1 coal seam: (**a**) safety-line method for gas pressure; (**b**) stepwise pattern for gas content.

The gas pressure and content of the 6-1 coal seam in the mining area were statistically analyzed to predict the gas occurrence trend. The safety-line method, for the prediction of gas pressure, has been applied in numerous coal mines in China [44]. As shown in Figure 15a, two symbol points of gas pressure were used to draw a safety-line for the prediction of the trend of gas pressure. When the burial depth reached 930 m, the gas pressure tended to exceed the critical values of 0.74 MPa. In Australia, the stepwise pattern is used to describe the increasing of the gas content with the burial depth [45]. Figure 15b shows the stepwise changes of the gas content at burial depths from 770 m to 780 m. When the mining depth reached 770~780 m, supplementary testing work to determine the gas content provided more precise prediction results. If the intervals of the strata were stable, the investigation results, including mining effect and gas control method, could be applied to new panels in the mining area, such as the 360802 face (Figure 1). The treatments for 6-1 coal were effective in reducing the gas content and the parameters of the crossing holes could be optimized for the next mining face, such as the interval of boreholes (*l*_1_ and *l*_2_ in Figure 4). This means that less drilling work could achieve the expected effect. This field investigation is thus beneficial in terms of the planning of gas control in mining areas.

## 6. Conclusions

Aiming at the control of gas disasters in new deep-mining areas, this investigation adopted the methods of theoretical analysis, numerical simulation and field testing to study the control model for safety production. The main conclusions drawn from the investigations are summarized below:

(1) The regularity of permeability within the protected layer was verified spatially by means of coal deformation and stress evolution. The maximum compressional and expansional deformation of the 6-1 coal were 18‰ and 28‰, respectively. The gas-permeability coefficient of the 6-1 coal underwent a decrease, an increase and finally stabilized. The high-permeability-coefficient area presented an “O-shape” distribution in the coal seam, and the maximum value was 55.8 m^2^/(MPa^2^·d).
(2) Applying gas disaster control and prevention treatments to the 360804 mining face produced significant protective effects on the underlying No. 6-1 coal seam. The residual gas content and pressure in the area, determined by the dip-pressure-relief angle of 78° and the strike-pressure-relief angle of 58°, were reduced to far less than the threshold values. The treatments, utilizing the mining effect and adopting the pre-drainage technology of crossing holes, played a very effective role in reducing the gas emission in the mining face and eliminating the outburst risk of 6-1 coal.
(3) The procedures, methods and results of the investigation are instructive for subsequent applications in other work faces with similar geological conditions. Reasonable prediction of gas occurrence can further improve the guiding role of the investigation results. All of this adds up to a safety strategy that is beneficial to the planning of gas control in successive panels.

## Figures and Tables

**Table 1:** Gas parameters of coal seam.

Coal Seam	Thickness, m	Gas Pressure, MPa	Gas Content, m^3^/t	Coal Hardiness Coefficient	Initial Velocity of Diffusion of Coal Gas
No. 6-1	2.65	0.48	4.23	0.37	9
No. 7	2.52	0.3	2.45	0.37	6
No. 8	3.34	0.4	3.51	0.27	7
No. 9	1.3	0.4	3.28	0.37	6

**Table 2:** Parameters of gas emission calculation.

Parameters	Value
Rock emission coefficient, $K_{1}$	1.3
Coal drop coefficient, $K_{2}$	1.08
Preparatory workings discharge coefficient, $K_{3}$	0.86
Mining height, M, m	3
Original gas content of No. 8 coal, $w_{0}$, m^3^/t	3.51
Residual gas content of No. 8 coal, $w_{c}$, m^3^/t	0.63
Original gas content of No. 6-1 coal, $w_{01}$, m^3^/t	4.23
Residual gas content of No. 6-1 coal, $w_{c1}$, m^3^/t	0.74
Seam thickness, *m*, m	3
Emission ratio of no. 6-1 coal, $\eta_{1}$	0.25
Original gas content of No. 7 coal, $w_{02}$, m^3^/t	2.45
Residual gas content of no. 7 coal, $w_{c2}$, m^3^/t	0.55
Emission ratio of No. 7 coal, $\eta_{2}$	0.5
Original gas content of No. 9 coal, $w_{03}$, m^3^/t	3.28
Residual gas content of No. 9 coal, $w_{c3}$, m^3^/t	0.62
Emission ratio of No. 9 coal, $\eta_{3}$	0.9

**Table 3:** Mechanical parameters of coal and rock.

Lithology	Density, kg/m^3^	Bulk Modulus, GPa	Shear Modulus, GPa	Cohesion, MPa	Friction, °
Quartz sandstone	2530	13.67	9.41	6.22	34.1
Fine sandstone	2550	8.05	24.79	6.3	36
Mudstone	2100	0.24	0.16	2.3	32
Sandy mudstone	2600	13.67	9.41	6.12	30
Coal	1400	0.23	1.91	0.3	32
Goaf material	1000	0.45	0.60	0	40

**Table 4:** Parameters for strain-softening model.

Volume Strain/%	Friction/°	Cohesion/MPa
0	35	0.4
0.03	20	0.2
0.1	10	0.01

**Table 5:** Parameters for double-yield model.

Volume Strain, %	Cap Pressure, MPa	Volume Strain, %	Cap Pressure, MPa
0	0.01	0.12	8.5
0.02	0.2	0.14	11.5
0.04	0.8	0.16	19
0.06	1.3	0.18	34
0.1	5.5	0.2	50

**Table 6:** Test of residual gas pressure and content for 6-1 coal seam.

No.	Site	Gas Content, m^3^/t	Gas Pressure, MPa
1	360604 floor auxiliary roadway (in Figure 1 site A)	1.97	0.1
2	360604 floor auxiliary roadway (in Figure 1 site A)	--	0.1
3	360604 floor auxiliary roadway (in Figure 1 site A)	2.01	0.1
4	360604 floor roadway (in Figure 1 site C)	2.01	0.1

## Data Availability

Not applicable.
